# Addressing Anharmonic Effects with Density-Fitted Multicomponent Density Functional Theory

**DOI:** 10.1021/acs.jpca.5c00382

**Published:** 2025-04-07

**Authors:** Lukas Hasecke, Maximilian Breitenbach, Martí Gimferrer, Rainer Oswald, Ricardo A. Mata

**Affiliations:** Institute of Physical Chemistry, University of Göttingen, Tammannstrasse 6, 37077 Göttingen, Germany

## Abstract

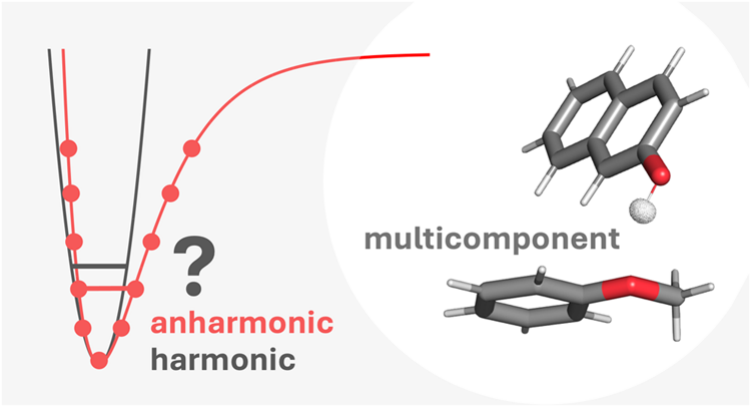

In this contribution we present the first local density-fitted multicomponent density functional theory implementation and assess its use for the calculation of anharmonic zero-point energies. Four challenging cases of molecular aggregates are reviewed: deprotonated formic acid trimer, diphenyl ether-*tert*-butyl alcohol conformers, anisole/methanol and anisole/2-naphtol dimers. These are all cases where a mismatch between the low-temperature computationally predicted minimum and the experimentally determined structure was observed. Through the use of nuclear-electronic orbital energies in the thermodynamic correction, the correct energetic ordering is recovered. For the smallest system, we compare our results to vibrational perturbation theory anharmonically corrected zero-point energy, with perfect agreement for the lower-lying conformers. The performance of the newly developed code and the density fitting errors are also analyzed. Overall, the new implementation shows a very good scaling with system size and the density fitting approximations exhibit a negligible impact.

## Introduction

Albeit being introduced for several dozens of years,^1−4^ multicomponent methods have yet to find widespread usage and an established position in the computational chemist toolbox. These are a unique class of quantum chemical methods, whereby electrons and a selected set of other Fermions (most commonly protons) are handled on the same footing. In the case of Hartree–Fock and Density Functional Theory (DFT), this is achieved by building a total Fermionic wave function as a product of both systems wave functions. From this point onward, and for ease of discussion, we will restrict ourselves to the treatment of protons. The multicomponent methodology entails a partial lifting of the Born–Oppenheimer approximation, with selected protons being described through a wave function. The solution for the protonic subsystem is coupled with the electronic subsystem. The coupling is determined by the level of theory applied, foremost through the Coulomb interactions but potentially by refined approaches to electron–proton correlation. One of the most popular denominations for the methods is the nuclear-electronic orbital (NEO) theory,^5,6^ which we also use to denominate our implementations.

When it comes to practicality, DFT is clearly the framework of choice for several reasons. With the simplicity of a mean-field theory one is still able to handle correlation explicitly through parametrized exchange and correlation functionals. In the case of NEO–DFT,^7−9^ this involves solving the Kohn–Sham equations for two different effective potentials
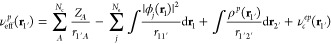
1
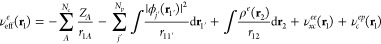
2whereby the indices *A* stand for *N*_*c*_ classical nuclei, *j* for the *N*_*e*_ electrons and the dashed indices for the *N*_*p*_ quantum protons. The potentials and densities are marked with *e* and *p* accordingly. In the case of the effective potential ν_eff_^*p*^ felt by protons, one has the interaction with classical nuclei, electrons, other quantum protons and the electron–proton (ν_*c*_^*ep*^) correlation potential. The electrons and quantum protons couple through the Coulomb interaction and electron–proton correlation. The electron–electron correlation bears the same form as in Born–Oppenheimer calculations.

As one can observe in the equations above, one needs only to provide a functional for electron–electron exchange-correlation (ν_*xc*_^*ee*^) and for electron–proton correlation (ν_*c*_^*ep*^). This is because both proton–proton exchange and correlation are negligible for chemically relevant systems. In our implementation, in order to exclude self-interaction, the diagonal elements of the Hartree–Fock exchange term are added to the protonic effective potential.^10^ In comparison to standard electronic structure theory, this only leaves the definition of the electron–proton correlation functional unresolved. When the distinct characteristics of both Fermions are accounted for, correlation functionals derived from first-principles can be applied to multicomponent DFT.^11^ In the context of this work, however, we will stray away from the discussion of density functional development and restrict ourselves to the use of one of the most widely used functionals, the epc-17.2 functional proposed by Hammes-Schiffer and co-workers.^12^ Their expression is derived analogous to the Colle-Salvetti formalism^10,13^ and is provided as
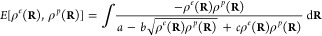
3The functions ρ^*e*^(**R**) and ρ^*p*^(**R**) represent the electronic and nuclear densities at the real space position denoted by **R**.^10^ The parameters *a* = 2.35, *b* = 2.40 and *c* = 6.6 are chosen according to the functional formulation in ref (12). We have used the same development framework in Molpro^14^ as for our previously published local density-fitted NEO-HF program.^15^ The most relevant feature, next to the local density fitting within the electronic system, in the case of NEO–DFT is that density fitting approximations are used for the electron–proton Coulomb coupling. In general, the density fitting approximation is reducing the computational effort of calculating the four-center two-particle integrals as
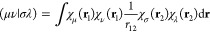
4consisting of atom centered basis functions {χ_μ_}, which are now within this approximation expressed in terms of 2-index Coulomb metrics
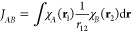
5and 3-index integrals
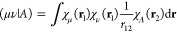
6where {χ_*A*_} are denoting the necessary auxiliary fitting basis functions. The respective densities are then approximated by a linear combination of those auxiliary density fitting functions
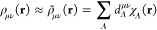
7The indices μ and ν refer in general basis functions, either electronic or nuclear. The fitting coefficients are determined through a so-called robust fitting as described previously.^15−17^ Regarding the performance of the computational protocol several in-depth studies were already carried out to assess the computational efficiency of the local and canonical density fitting approximations in a multicomponent method context.^15,18−21^ Thus, the main objective of this work is to demonstrate how this computationally efficient implementation of NEO–DFT can be applied routinely to reliably estimate anharmonic effects in the zero-point vibrational energy of hydrogen-bonded molecular clusters. One should note that the systems chosen are not close to the limit of our application range. The same benchmark systems as used in ref (15) can be routinely computed.

## Computational Details

All NEO calculations have been carried out with Molpro,^14^ employing the B3LYP functional including the D3 dispersion correction with Becke-Johnson damping.^22,23^ The (local) density-fitted version of the NEO–DFT module integrated in Molpro is an extension of our previously presented (local) density-fitted NEO restricted HF implementation.^15^ Thereby, we employ (local) density fitting within the electronic subsystem and for the Coulomb coupling between quantum mechanical treated nuclei and electrons. The subsystem of the quantum nuclei is treated by an integral-direct implementation.^15,18^ Overall, the general implementation together with accuracy and performance of density fitting within multicomponent DFT was previously discussed by Mejía-Rodríguez et al.^21^ For the quantum mechanical nuclei the PB4-F2 nuclear basis set together with the even tempered 10s10p10d10f fitting basis set with exponents ranging from 2√2 to 64 are employed.^24^ Both NEO and regular DFT calculations are carried out employing the def2-TZVP, or def2-TZVPP in the case of the deprotonated formic acid trimer, basis sets with the def2-QZVPP-JKFIT density fitting basis set.^25,26^ A threshold of 10^–7^ a.u. for the energy difference within the electronic and nuclear SCF computations, the difference in the density between iterations and the gradient of the respective nuclear and electronic subiterations. The overall energy difference in the NEO–DFT iterations was set to a threshold of 10^–6^ Hartree. All Molpro computations employ the direct inversion in the iterative subspace starting after the first iteration with a maximum of 10 Fock matrices as basis to extrapolate.^27,28^ In general the standard grid 3 is employed for the computations, whereas the formic acid clusters are computed with the standard grid 2.^29^ The electron–proton correlation is computed with the epc-17.2 functional.^12^ In order to assess the error of the density fitting, reference calculations for the formic acid clusters with regular NEO–DFT are carried out with Q-Chem 6.2.^30^ Thereby, the standard grid 2, a threshold of 10^–8^ Hartree for the energy and the geometric direct minimization algorithm were employed.^29,31^ For those systems also the threshold within Molpro was raised to 10^–8^ a.u. for the energy difference within the NEO microiterations and 10^–7^ for the overall energy difference. These tighter thresholds were also employed for the NEO-RHF reference wave function of the NEO(MP2)-PNO-LCCSD(T)-F12 method.^18^ Those calculations employ the cc-pVTZ-F12 basis set with the cc-pVQZ-JKFIT density fitting basis for the Fock and the exchange matrices as well as the complementary auxiliary basis set for the resolution of the identity and the cc-pVQZ-MP2FIT density fitting basis set.^32−34^ The F12b energies are obtained with the 3*A(LOC,FIX) ansatz. Moreover, the complementary auxiliary basis set singles correction together with the scaling of the perturbative triples are applied.^35−38^ All systems are optimized with Gaussian16 employing the def2-TZVP basis set with very tight SCF settings, tight optimization thresholds and a superfine grid utilizing B3LYP-D3(BJ).^39^ In the case of the deprotonated formic acid trimer the def2-TZVPP basis set is employed.^25^ Corresponding frequency calculations are carried out with the same settings. In order to obtain the zero-point vibrational energies (ZPVE) of the systems without the contributions of the quantum mechanical treated protons, the isotope mass of the respective proton centers was set to 9.9 × 10^12^ a.u., making those protons infinitely heavy. Those ZPVEs corrections without the quantum nuclei contributions are used for the NEO computations, whereas the full ZPVEs are applied as corrections to the regular Born–Oppenheimer based computations. The second-order vibrational perturbation theory calculations were also carried out with Gaussian16. For these specific calculations particularly tight thresholds were required for both optimization and the numerical integration grid (keywords very tight and superfine, respectively). All nuclear densities shown are displayed at a 0.02 σ contour level generated with the PyMOL 2.5.2 program.^40^

## Results and Discussion

Inherently including nuclear quantum effects (NQEs) like anharmonic zero-point vibrational energy and delocalization within multicomponent DFT carries several advantages.^8,9^ These will be demonstrated on challenging examples whereby regular Born–Oppenheimer DFT is either providing inaccurate or mismatching results. For our study we chose the B3LYP-D3(BJ) electronic functional, the most commonly used functional to date for organic compounds.^22,41^ All of the experiments later mentioned in the text approach the very-low-temperature limit and near-vacuum conditions (or matrix when explicitly noted) so we will always be comparing the latter to 0 K computed enthalpies.

### Deprotonated Formic Acid Trimers

**Figure 1:**
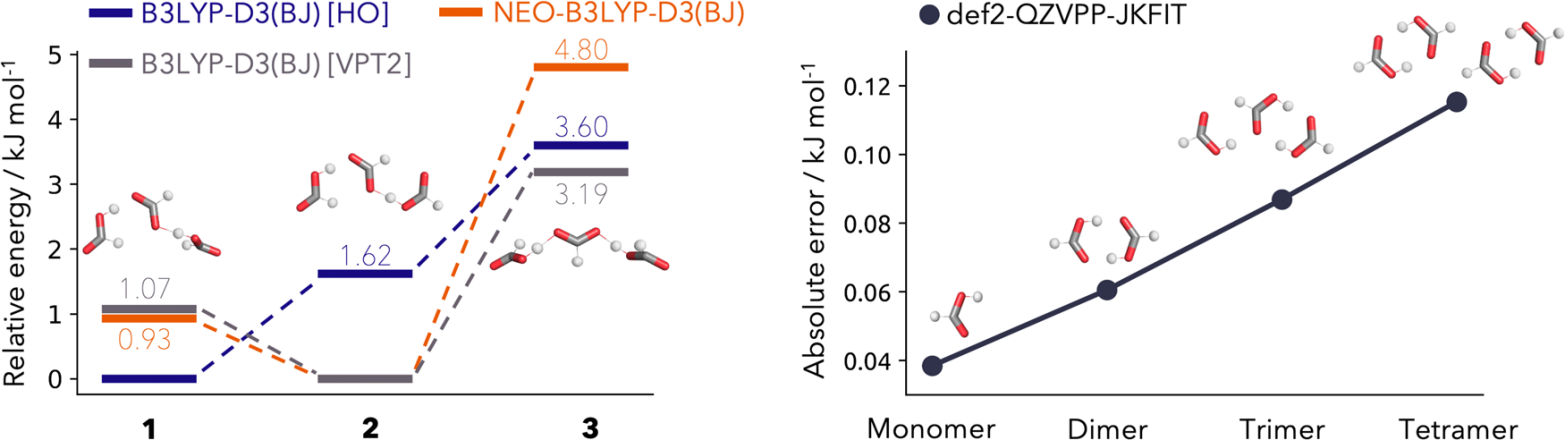
Left: Relative energies of the deprotonated formic acid trimer isomers computed with regular B3LYP-D3(BJ) (blue), NEO-B3LYP-D3(BJ) (orange) and anharmonically corrected via vibrational perturbation theory (gray) utilizing the def2-TZVPP electronic and PB4-F2 nuclear basis set. Right: Absolute error of the density-fitted NEO-B3LYP-D3(BJ) method computed with the def2-QZVPP-JKFIT density fitting basis set in comparison to the regular NEO-B3LYP-D3(BJ) results for size increasing formic acid clusters utilizing the def2-TZVP electronic and PB4-F2 nuclear basis set.

The first example is the deprotonated formic acid trimer which was thoroughly analyzed via infrared action spectroscopy in helium nanodroplets by Taccone et al.^42^ Three conformers of interest were identified (see Figure 1). One finds a mismatch between the prevalent structure observed in experiment and the computed global minimum. The potential reasons for this mismatch include the harmonically computed zero-point vibrational energy, as well as potential shortcomings of the experiment, namely kinetics and dynamics of the cluster formation and solvent effects.^42,43^ In the closely related proton-bound formate dimer system, constrained NEO molecular dynamics have already demonstrated the usefulness of multicomponent calculations in such strongly bound hydrogen-bond systems.^44^ In Figure 1 we show the resulting energetic orderings of the three lowest isomers obtained with Born–Oppenheimer based harmonically corrected B3LYP-D3(BJ), anharmonically corrected via vibrational perturbation theory (VPT2) and finally multicomponent NEO-B3LYP-D3(BJ). The fundamental bands of the individual conformers are given in Table S1. In general, the harmonic B3LYP-D3(BJ) results are in agreement with the ordering reported by Taccone et al.^42^ One should note that in their study not only density functional based but also wave function methods, MP2 and CCSD(T), as well as double-hybrid approaches result in the same energetic ordering.^42^ The latter is in disagreement with both VPT2 and NEO results. Instead of isomer **1**, isomer **2** becomes the global minimum by 1.07 and 0.93 kJ mol^–1^ for VPT2 and NEO, respectively. These are in agreement with the experimental exclusive observation of isomer **2**. The main disagreement between the VPT2 and the NEO value is found for isomer **3**. NEO–DFT further destabilizes isomer **3** by 1.20 kJ mol^–1^ compared to the harmonic B3LYP-D3(BJ) result. The VPT2 correction actually stabilizes isomer **3** by 0.41 kJ mol^–1^. Reviewing the VPT2 calculations it is worth noting that extremely tight criteria for optimization (and respectively for the DFT numerical grids) have to be used. Otherwise one risks variations in the relative zero-point vibrational energies of a few kJ/mol. But even with very tight thresholds VPT2 struggles to describe anharmonic O–H bonds, since it is based on a quartic force field built with local information.^45^ This is particularly serious for isomer **3**, where the combined symmetric stretching mode of the two bridging protons is red-shifted from its harmonic value by 733 cm^–1^ (2535 cm^–1^ vs 1802 cm^–1^) and exhibits a low overlap with the harmonic mode. This could be the reason for the discrepancy observed in the last isomer.

In general, it is worthwhile to analyze the obtained results in light of the different hydrogen bonding patterns of the three isomers. Isomer **2**, which is the global minimum on the NEO and anharmonic corrected PES, exhibits with four intermolecular hydrogen bonds the most intertwined isomer. Besides the −OH ··· O– and two −CH ··· O– hydrogen bonds it also exhibits a −O ··· H ··· O– interaction with a more shared character, where the position of the hydrogen is shifted by around 0.04 Å further toward the center of the bond as the other isomers. The global minimum on the NEO and the anharmonic corrected PES is the isomer **2**. This isomer exhibits only three hydrogen-bond interactions between the molecules. It has two regular −OH ··· O– and one −CH ··· O– type interactions. Overall it lacks one hydrogen-bond interaction compared to isomer **2** and does not show any shared hydrogen character. This could be a possible justification why this aggregate is less stable than isomer **2**, which exhibits more stabilizing interactions between the individual molecules. Following this trend isomer **3** has the least amount of stabilizing hydrogen-bond interactions, only two −OH ··· O– type interactions, and thus is the least stable isomer in line with all observations.

We now turn to the potential numerical errors introduced by the density fitting approximations used.^14,21^ In order to verify the validity of our results we have to benchmark the errors on absolute and relative energies. Therefore, we first employ size increasing clusters of the formic acid molecule ranging from one up to a cluster of four molecules. The obtained results are displayed in Figure 1. The absolute error introduced by the density fitting scales very low in regards to the overall system size. For the formic acid monomer the absolute error is only 0.04 kJ mol^–1^ and increases only slightly up to 0.12 kJ mol^–1^ for the formic acid tetramer. The absolute error of the trimer, which is slightly bigger than its deprotonated counterpart, is only 0.09 kJ mol^–1^. In order to also assess the impact of the density fitting on the relative energies we analyzed the differences in the energies for density-fitted B3LYP-D3(BJ) and regular B3LYP-D3(BJ) results. The obtained energies are shown in Table S1. The overall root-mean-square deviation between the relative energies is only 0.001 kJ mol^–1^. Such a minor error on relative energies is widely expected.^35−37,46^ In general, the error introduced by density fitting approximations, especially for relative energies, seems negligible.

### Diphenyl Ether-*tert*-butyl Alcohol Dimers

**Figure 2:**
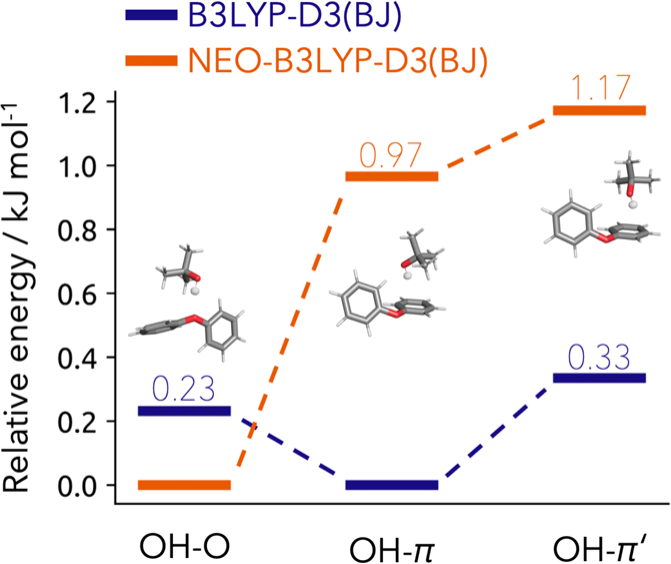
Relative energies of the diphenyl ether-*tert*-butyl alcohol isomers computed with regular B3LYP-D3(BJ) (blue) and NEO-B3LYP-D3(BJ) (orange) utilizing the def2-TZVP electronic and PB4-F2 nuclear basis set.

The next system we would like to revisit with NEO–DFT is the diphenyl ether–*tert*-butyl alcohol complex. The complex was investigated with a multispectroscopic approach by Bernhard et al.^47^ This included FTIR spectroscopy, IR/UV spectroscopy and chirp pulse Fourier transform microwave spectroscopy together with a broad mixture of theoretical approaches going from B3LYP-D3(BJ), over MP2 and CC2 calculations to symmetry adapted perturbation theory.^47^ Experimentally the results point in the direction that the OH-O bound dimer is slightly more stable than the OH-π bound isomer. This was derived based on almost similar abundance resulting from the FTIR spectra from the helium expansion, the higher abundance of the OH-O isomer in the mass- and isomer-selective IR/R2PI spectra in neon expansion supported by the broadband rotational spectra in helium and neon.^47^ In general the experimental preference toward the OH-O bound dimer was estimated to be in the energy range of 0–1 kJ mol^–1^. Theoretical results however vary largely for the methods employed in their analysis.^47^ We recomputed the most stable isomers, predicted by the previous work, with regular B3LYP-D3(BJ) and NEO-B3LYP-D3(BJ). The obtained results are shown in Figure 2. In agreement with prior B3LYP-D3(BJ) results the most stable isomer is predicted to be OH-π bound. It is a rather small energetic gap to the OH-O bound isomer with only 0.23 kJ mol^–1^, which would be in agreement with the experimental observation of both species. Moreover, the second determined OH-π′ structure is energetically 0.33 kJ mol^–1^ higher than the OH-π structure which again is in agreement with experiment. By rotational spectroscopy the OH-π structure was assigned to be the observed species instead of OH-π′.^47^ However, B3LYP-D3(BJ) predicts the OH-π bound dimer to be energetically preferred over the OH-O bound dimer which is against the experimental conclusions. By employing NEO–DFT the ordering of the PES changes. The global minimum obtained from the multicomponent PES is the OH-O bound dimer, whereas the OH-π bound dimer is energetically 0.97 kJ mol^–1^ higher in energy. Both observations are in agreement with the experimentally derived balance of the OH-O and OH-π bound dimer. In addition, the OH-π′ structure is energetically 0.20 kJ mol^–1^ higher as the OH-π structure, which agrees with the rotational spectroscopy experiment.

In this case one can also rationalize the obtained energetic ordering with an analysis of the bonding pattern. The most stable OH-O bound dimer exhibits one −OH ··· O– type interaction as well as one −CH ··· O– and one −CH ··· π– interaction. The OH-π bound dimers are rather similar in their −OH ··· π–, −CH ··· O– and −CH ··· π– interaction patterns. However, the angle between the −C=C– and bonding hydrogen atom is different for both systems. While the −CH ··· π– interaction exhibits an angle of 76° and is centered on the middle of the −C=C– bond, the OH-π′ dimer has an angle of 85° and is tilted toward the α-carbon atom. While those different π-bound isomers are both correctly energetically differentiated by both regular Born–Oppenheimer as well as NEO methods, only the change of stability between the OH-O and OH-π bound species remains open. The major difference of both isomers is the −OH ··· O– versus −OH ··· π– interaction. This subtitle balance of bonding types was already carefully analyzed with respect to various substrates for diphenyl ether by Dietrich et al.^48^ Experimentally, the diphenyl ether-*tert*-butyl system seems to energetically prefer the −OH ··· O–, whereas regular DFT underestimates either the −OH ··· O– interactions or is overestimating the −OH ··· π– interaction. Those sensitive balance and the origin of this difference will be elucidated in more detail within future work. Overall, the results obtained by NEO–DFT agree in all points with the experimental observations with minimal added computational effort.

### Anisole Methanol/2-Naphthol Dimers

**Figure 3:**
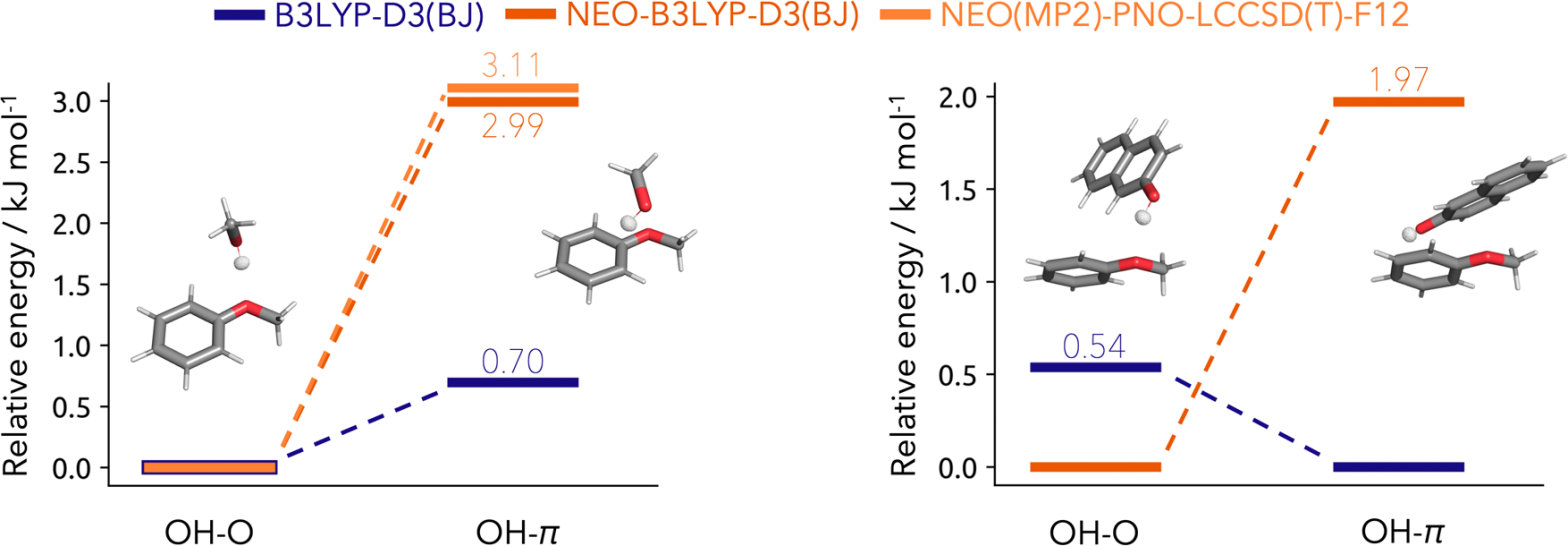
Left: Relative energies of the anisole-methanol dimer isomers computed with regular B3LYP-D3(BJ) (blue) and NEO-B3LYP-D3(BJ) (orange) utilizing the def2-TZVP electronic and PB4-F2 nuclear basis set together with the NEO(MP2)-PNO-LCCSD(T)-F12 (light orange) method utilizing the cc-pVTZ-F12 electronic and PB4-F2 nuclear basis set. Right: Relative energies of the anisole 2-naphthol dimer isomers computed with regular B3LYP-D3(BJ) (blue) and NEO-B3LYP-D3(BJ) (orange) utilizing the def2-TZVP electronic and PB4-F2 nuclear basis set.

We now turn to the last two reference systems. The employed examples are dimers formed by anisole with either methanol or 2-naphthol. Both dimers have already been extensively analyzed, both experimentally and theoretically.^49,50^ We start our discussion with the anisole-methanol dimer. The experimental analysis of this system is based on FTIR spectroscopy from a cold supersonic jet expansion in helium.^49^ They measured a 20 times lower abundance of the OH-π isomer than the preferred OH-O isomer. As a result they estimate the OH-π bound dimer to be at least 1 kJ mol^–1^ less stable than the OH-O dimer. The theoretical methods applied to this system give different results. In case of MP2 the OH-π dimer is clearly favored by 0.6 kJ mol^–1^, B3LYP-D3(BJ) favors the OH-O dimer by 0.7 kJ mol^–1^, the double-hybrid B2PLYP favors the OH-O dimer by 1.1 kJ mol^–1^ and CCSD(T) indicates an almost isoenergetic balance between the two.^49^ In general they found, that the balance could be further shifted toward the OH-O bound dimer by 0.2 kJ mol^–1^, if the aug-cc-pVTZ basis set instead of the def2-TZVP basis set is employed for the geometry optimization and energy computation.^49,51,52^ However, the dependence of the initial geometry is dramatically reduced by employing multicomponent methods since the quantum particle will instantaneously adapt to its preferred position. This was already demonstrated in a thorough benchmark for the hydrogen bound methanol complexes with different furan derivatives.^18^ Therefore, we first compare the B3LYP-D3(BJ), NEO-B3LYP-D3(BJ) as well as NEO(MP2)-PNO-LCCSD(T)-F12^18^ results on the basis of the B3LYP-D3(BJ)/def2-TZVP optimized structures. The NEO(MP2)-PNO-LCCSD(T)-F12 method extends the computation of the electronic correlation energy to local coupled cluster with singles and doubles excitations and perturbative triples, whereas the nuclear correlation as well as the nuclear-electronic correlation are obtained by utilizing second-order Møller–Plesset perturbation theory.^18^ The obtained results are displayed in Figure 3. The regular B3LYP-D3(BJ) method leads to a preference toward the OH-O structure with 0.7 kJ mol^–1^ compared to the OH-π bound dimer. This is in agreement with the results obtained by Heger et al.^49^ Utilizing NEO-B3LYP-D3(BJ) and NEO(MP2)-PNO-LCCSD(T)-F12 instead of regular B3LYP-D3(BJ) carries several advantages as mentioned before. First of all the dependency of the initial geometry is reduced, allowing for computational efficient optimization with small basis sets.^18,53^ As a result, the NEO–DFT single-point calculation utilizing the def2-TZVP basis set already leads to experimental comparable results by favoring the OH-O bound dimer by 2.99 kJ mol^–1^. Compared to the NEO(MP2)-PNO-LCCSD(T)-F12 method, which includes explicit correlation in order to achieve almost complete basis set results, the NEO–DFT method performs very similarly. Given an absolute energy difference of only 0.12 kJ mol^–1^ between both methods, NEO–DFT seems to perform very well on this system. Moreover, the NEO–DFT exhibits favorable computational effort to achieve the result compared to the higher level approach. The NEO(MP2)-PNO-LCCSD(T)-F12 was running with a total CPU time of 193 min to compute both systems, compared to 12 min for the NEO-B3LYP-D3(BJ) calculations.

In order to elucidate the basis set dependence of the DFT based results, we computed the energy balance of the anisole-methanol dimer for different basis sets as shown in Table S3. Thereby, the geometry optimization, single-point energy calculation and zero-point vibrational energy correction are computed all at the same level of theory. Our harmonically corrected results are in agreement with the values provided by Heger et al.^49^ With increasing basis set size, the OH-π bound dimer relative energy is estimated at about 1 kJ mol^–1^. The experimental observations would be more in line with an energy difference of 2 kJ mol^–1^ or more.^49^ Our multicomponent approach placed the relative energy at about 3.5 kJ mol^–1^.

We would like to emphasize the advantages of NEO–DFT also for the next test system, the dimer formed between anisole and 2-naphthol. Experimentally a combination of jet-cooled FTIR spectroscopy and laser-induced fluorescence spectroscopy, as well as resonance-enhanced two-photon UV ionization spectroscopy was employed by Nejad et al.^50^ They observed only the OH-O bound dimer, whereas the OH-π bound dimer remains experimental elusive.^50^ The theoretical methods applied also provide diverse results from B3LYP-D3(BJ) favoring the OH-π structure by 0.7 and 0.5 kJ mol^–1^ for the def2-TZVP and def2-QZVP basis sets and only twist the preference slightly to the OH-O structure with 0.1 and 0.2 kJ mol^–1^ for the def2-TZVP and def2-QZVP basis sets if three-body dispersion corrections are included.^50^ However, neither of those results would energetically explain the complete elusiveness of the OH-π dimer. Moreover, unscaled MP2 variants also favor the OH-π structure. By employing SCS-MP2 or PNO-LCCSD(T)-F12b the OH-O structure is favored.^50^ PNO-LCCSD(T)-F12b/cc-pVTZ-F12 should be within a kJ mol^–1^ of the complete basis set limit of CCSD(T),^32,36,37^ the current gold standard of quantum chemistry. In this case, the OH-O structure is about 3 kJ mol^–1^ lower in energy. Overall, this provides an excellent benchmark for the NEO-DFT method. The results obtained for the anisole 2-naphthol dimer with regular B3LYP-D3(BJ) and NEO-B3LYP-D3(BJ) are displayed in Figure 3. B3LYP-D3(BJ) prefers the OH-π bound dimer by 0.54 kJ mol^–1^ which is in agreement with the results from Nejad et al.^50^ However, by employing NEO–DFT the PES changed noticeably to a strong preference of the OH-O bound dimer which is energetically favored by 1.97 kJ mol^–1^. Compared to the PNO-LCCSD(T)-F12b and the experimental conclusion, the obtained results with NEO-B3LYP-D3(BJ) are in line with both. However, it should be noted that the NEO–DFT results are obtained at much lower computational costs in comparison to the PNO-LCCSD(T)-F12b results.

## Conclusions

In total, we address theory-experiment mismatches in three cold molecular clusters systems, recovering the agreement between the computed and the measured energetic trends. For all cases addressed, the main reason for the disagreement could be traced back to the use of the harmonic approximation in calculating zero-point vibrational energies. A simple NEO single-point calculation proved enough to recover the right relative energetics. This is still only a partial correction, but which appears effective for a balanced treatment of such systems with varying degrees of hydrogen-bond strengths and proton delocalization.

This is a step forward in providing the right answer for the right reasons for challenging cases to regular DFT calculations. Therefore, it is particularly useful for the analysis of experiments targeting cold molecular clusters. In future studies, we will combine multicomponent methods and anharmonic vibrational calculations to observe in greater detail the limitations of both approaches, taking inspiration from the formic acid trimer results we have provided here.^54−56^ Moreover, incorporating additional experimentally determined benchmark observables, such as the dipole moments of these aggregates, would be valuable for further elucidating the advantages of multicomponent methods and establishing a foundation for future method development.

## Data Availability

Structural information and energies are available free of charge on GRO.data (10.25625/2IPVNS).

## Supporting Information Available

Fundamental bands of the deprotonated formic acid trimers Table S1, relative energies of the deprotonated formic acid trimers with respect to the energetically most stable isomer 1 Table S2, relative energies of the anisole-methanol OH-π bound isomer with respect to the OH-O bound isomer for different basis sets Table S3 (PDF)
