# Supplementary material for: Addressing Anharmonic Effects with Density-Fitted Multicomponent Density Functional Theory
Source: J Phys Chem A. 2025 Apr 7;129(15):3560–6. doi: 10.1021/acs.jpca.5c00382 (PMC12010317; doi:10.1021/acs.jpca.5c00382)
Supplement: Supplementary file 1 — jp5c00382_si_001.pdf [file jp5c00382_si_001.pdf]

Supporting Information: Addressing Anharmonic Effects with Density-Fitted Multicomponent Density Functional Theory

Lukas Hasecke,* Maximilian Breitenbach, Martí Gimferrer, Rainer Oswald, and Ricardo A. Mata*

Institute of Physical Chemistry, University of Göttingen, Tammannstrasse 6, 37077, Göttingen, Germany

E-mail: lhaseck@gwdg.de; rmata@gwdg.de

Table S 1: Calculated fundamental bands (in cm^{-1}) for the 3 conformers of the deprotonated formic acid trimer. The level of theory used was B3LYP-D3(BJ)/def2-TZPP. The results include harmonic frequencies as well as VPT2 anharmonic values. All values in this table were calculated with the Gaussian16, Revision A.03 program package, using `VeryTight` thresholds for optimization and `SuperFine` option for the numerical integration grid. In cases where the anharmonic state shows a small overlap to the respective harmonic mode, the anharmonic value is italicized. As one can observe, such a mismatch is observed for the H-stretch bands only in Conformer **3** (which also shows issues in the low-frequency region). The H-stretches are labeled, also including information if two bonds are coupled (in those cases, s: symmetric and a: antisymmetric).

Conformer **1**			Conformer **2**			Conformer **3**		
	harm	anharm		harm	anharm		harm	anharm
νCH	2985.0	2854.5	νCH	2985.6	2852.7	νCH/CH(s)	2928.1	2691.1
νCH	2927.6	2699.0	νCH	2946.5	2828.2	νCH/CH(a)	2927.5	2701.2
νCH/OH(s)	2876.0	2688.0	νCH	2916.4	2770.0	νCH	2852.7	2652.6
νCH/OH(a)	2818.4	2334.9	νOH	2751.4	2290.9	νOH/OH(s)	2535.1	*1802.1*
νOH	2484.8	1762.3	νOH	2003.1	1135.8	νOH/OH(a)	2461.5	1725.4
	1797.6	1760.1		1789.6	1749.0		1797.8	1749.4
	1793.1	1746.0		1734.4	1632.4		1797.7	1759.8
	1648.1	1576.4		1573.3	1304.7		1642.1	1554.8
	1522.6	1474.8		1562.5	1528.4		1527.1	1473.0
	1494.8	1442.4		1500.2	1438.4		1525.8	1470.4
	1428.5	1395.3		1428.2	1394.9		1421.8	1409.9
	1421.7	1392.9		1412.6	1381.5		1421.6	1378.7
	1410.5	1377.6		1405.9	1362.8		1411.6	1378.3
	1379.8	1352.8		1345.5	1208.8		1394.6	1364.3
	1244.0	1226.3		1292.1	1276.6		1245.2	1250.5
	1231.4	1283.1		1241.9	1237.0		1244.4	*1223.4*
	1215.9	1112.8		1235.1	1209.8		1211.2	1151.8
	1094.0	1104.3		1108.9	1120.3		1206.3	1186.8
	1079.2	1071.0		1090.4	1065.2		1079.0	1046.0
	1065.4	1046.0		1078.8	1011.9		1065.2	1023.8
	1063.8	*963.6*		1056.3	1041.1		1065.2	1074.5
	789.7	785.3		797.6	803.0		804.9	803.1
	711.2	704.4		734.9	735.7		710.6	700.1
	710.8	700.3		711.8	704.1		710.4	696.8
	302.6	297.4		326.8	362.8		326.0	313.5
	264.0	250.0		268.5	256.8		242.8	229.8
	203.7	205.5		227.6	216.6		174.3	*240.7*
	168.2	157.7		203.3	192.3		164.2	*160.6*
	153.3	*151.9*		185.6	182.9		142.4	212.7
	139.0	*92.6*		147.0	132.2		140.3	143.3
	131.6	135.4		140.0	135.6		115.0	*-41.1*
	96.9	*142.9*		81.3	76.7		95.3	*-8.0*
	49.2	*66.1*		62.1	59.5		55.4	52.6
	47.2	*25.2*		44.2	18.3		54.1	89.7
	35.2	12.1		42.0	29.2		19.2	*132.2*
	17.9	*20.6*		19.4	15.9		13.6	76.6

Table S2: Relative energies of the deprotonated formic acid trimers with respect to the energetically most stable isomer **1** given in kJ mol^{-1} for the regular B3LYP-D3(BJ) method and its density fitted counterpart.

Method	**2**	**3**
B3LYP-D3(BJ)	1.6207	3.5999
DF-B3LYP-D3(BJ)	1.6190	3.6000

Table S3: Relative energies of the anisole-methanol OH-π bound isomer with respect to the OH-O bound isomer for different basis sets computed with regular B3LYP-D3(BJ) and NEO-B3LYP-D3(BJ) given in kJ mol^{-1}.

Basis set	B3LYP-D3(BJ)	NEO-B3LYP-D3(BJ)
def2-TZVP	0.70	2.99
def2-TZVPP	0.76	3.27
aug-cc-pVTZ	0.98	3.41
def2-QZVP	1.04	3.54
def2-QZVPP	1.04	3.54
cc-pVQZ	1.00	3.53
